# An usual case of bilateral deep venous thrombosis with associated pulmonary embolus

**DOI:** 10.1093/omcr/omac115

**Published:** 2022-11-24

**Authors:** Hannah Plumptre, Marilena Giannoudi, John Kurian

**Affiliations:** Bradford Royal Infirmary, Bradford, UK; Bradford Royal Infirmary, Bradford, UK; Bradford Royal Infirmary, Bradford, UK

## Abstract

Congenital absence of the inferior vena cava (IVC) triggers collateral vessel growth to drain the peripheries and abdominal organs. This causes venous stasis and increases the risk of deep vein thrombosis (DVT) and pulmonary embolism. Typically, patients with absent IVCs present before 30 years of age, with bilateral DVT symptoms triggered by intense exercise. The abnormality can remain undetected as computed tomography imaging is not usually performed. Due to the increased risk of clotting, these patients should be on life-long anticoagulation. Raising clinical awareness of this condition, to ensure appropriate investigations and treatment, is important.

## INTRODUCTION

Abnormally developed inferior vena cavae (IVCs) affect ~4% of the population, including variants such as agenesis, hypoplasia, duplication and interruption [1, 2]. Such anomalies can be isolated or accompanied by other congenital abnormalities, e.g. polysplenia, asplenia and dextrocardia [2].

The agenesis or absence of an IVC is rare, prevalence estimated 0.0005–1% [3]. Causes include embryological malformation and perinatal IVC thrombosis causing blockage and subsequent regression of the IVC [4–6].

An absent IVC necessitates collateral vessel formation to drain the abdominal organs and peripheries [7]. These inefficient vessels increase the patient’s risk of deep vein thrombosis (DVT), pulmonary embolism (PE) venous stasis and ulceration [8–12]. Identification of these patients is important to help reduce their risk of adverse thrombotic events.

## CASE REPORT

A 24-year-old man presented to hospital with leg and hip pain, commencing during high-intensity interval training, an activity he regularly undertook. The pain originated in his right leg, spreading to the left and both hips. Additionally, he reported 1 day history of dyspnoea worse on exertion and associated dizziness. He denied symptoms of cough, fever, haemoptysis or pleuritic chest pain. He reported intentional weight loss of 3.5 stone over 1 year.

His past medical history comprised of asthma. He took no regular medications and didn’t smoke. There was no reported family history. He works as a financial manager.

Clinical observations were heart rate 103, respiratory rate 20, blood pressure 126/69 and oxygen saturations 100% on room air. Heart sounds were normal and chest was clear. There was no palpable lymphadenopathy. His right calf was swollen compared with the left and tender on palpation. Coordination, power and sensation of both legs were intact.

**Figure 1:**
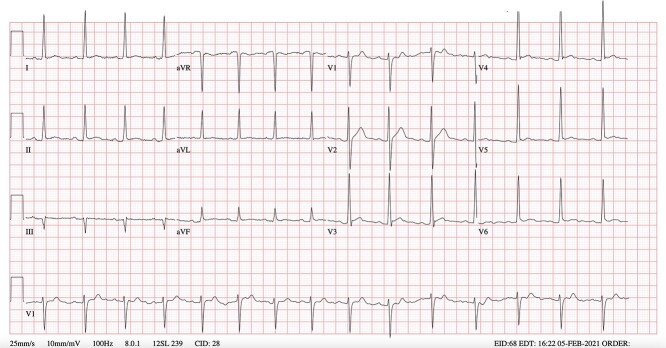
Patient’s admission ECG.

An electrocardiogram (ECG) revealed normal sinus rhythm and no cardiovascular strain (Fig. 1). A chest X-ray demonstrated an azygous fissure. Ultrasound of the right leg demonstrated a thrombotic occlusion of the great saphenous vein, from the right sapheno-femoral junction to the popliteal vein. Ultrasound of the left leg revealed a thrombus extending from the left common femoral vein into the popliteal vein. A handheld echocardiogram exhibited no right heart strain, and lab-measured troponin was 2.

**Figure 2:**
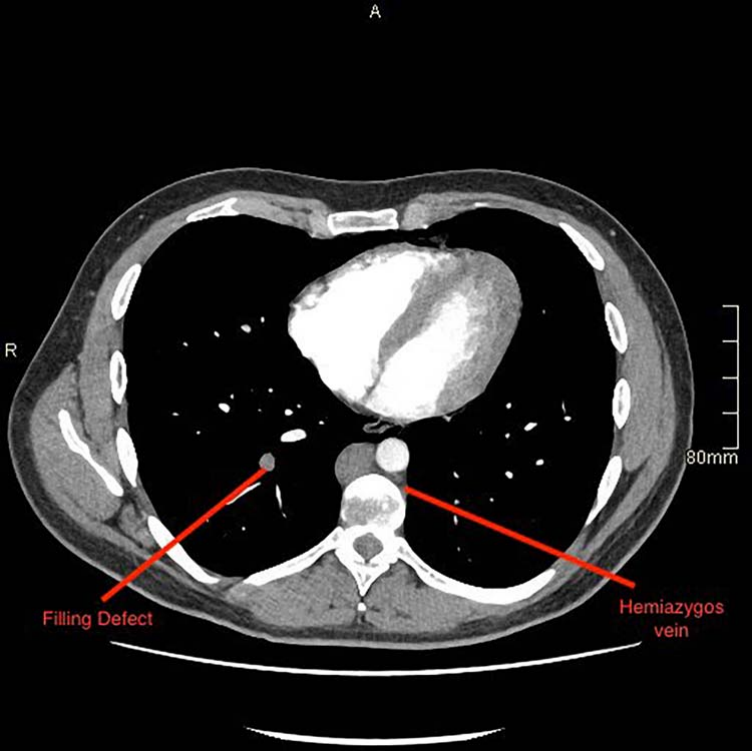
CTPA revealing pulmonary embolus within the right lung, labelled as filling defect.

**Figure 3:**
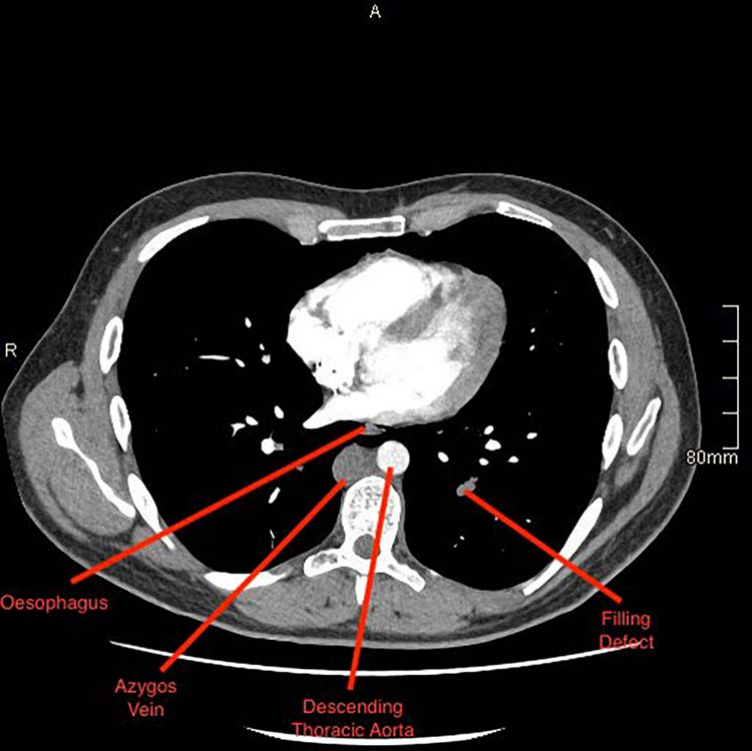
CTPA revealing pulmonary embolus within the left lung, labelled as filling defect.

**Figure 4:**
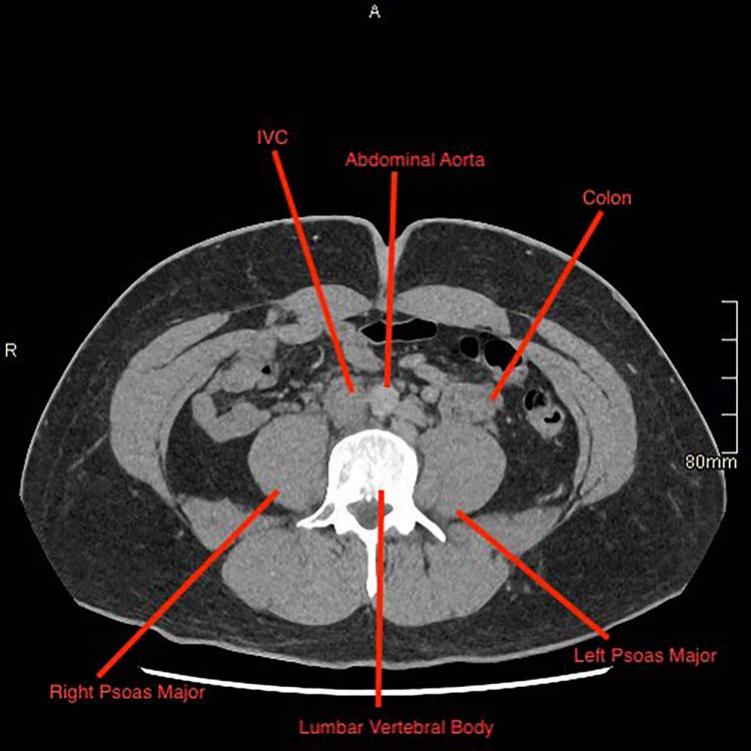
Sagittal CT image demonstrating the presence of the IVC.

**Figure 5:**
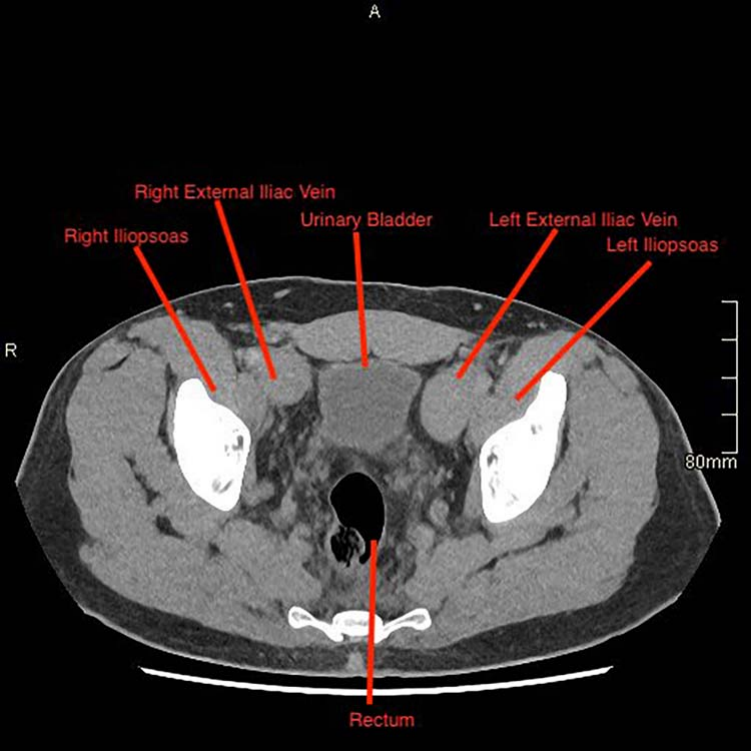
Sagittal CT image demonstrating an absent IVC.

A computed tomography (CT) pulmonary angiogram and thorax–abdomen–pelvis showed extensive bilateral PEs (Figs 2 and 3). There was no saddle embolus or radiological evidence of right heart strain. Prominent femoral and internal iliac veins were seen (Fig. 4). Clots were seen in both iliac veins and distal IVC. There was no IVC visible above the level of the renal veins (Fig. 5). The proximal IVC was patent, with venous collateralization in the retroperitoneum, suggesting a chronic diagnosis. Abdominal venous drainage was predominantly via the azygos and hemi-azygos system.

A thrombosis screen including; paroxysmal nocturnal haemoglobinuria, thrombophila screen, anti-nuclear antibody, antiphospholipid antibodies, beta-2-glycoprotein level, anticardiolipin levels, Hep B&C, human immunodeficiency virus screen and JAK-2 mutation screening was negative.

The gentleman was initially administered treatment-dose tinzaparin pending vascular MDT. Mechanical thrombectomy was considered but deemed not in his best interest due to the high risk of venous re-occlusion in the presence of persisting venous outflow obstruction. Lifelong anticoagulation was recommended. Given his haemodynamic stability throughout admission thrombolysis was deemed unnecessary. Anticoagulation with rivaroxaban caused rectal bleeding and he was switched to apixaban, which has been well tolerated. He remains asymptomatic at 1-year post-diagnosis, under follow-up from the haematology team.

## DISCUSSION

The IVC is made up of four parts, the hepatic, supra-renal, renal and infra-renal veins [13]. The phrase ‘absent IVC’ refers to an absence of all sections, or an absence of one or combinations of sections, with the mechanism best understood by looking at the embryological formation of the venous system. In utero, the vessels below the liver are formed by three pairs of veins, the subcardinal, posterior cardinal and supracardinal veins [13]. An absence of the entire IVC requires the incorrect formation of all three pairs. As the probability of this occurring as a systematic embryological error is unlikely, it is possible that an absent IVC without other congenital abnormalities may not be truly congenital, but rather pathological [14].

An absent IVC could be due to perinatal thrombosis [15]. One case describes a girl presenting with symptoms, and a consequent diagnosis of DVT within 48 hours of her birth, and no discernable IVC on ultrasound at that time [16]. When rescanned aged 12, a thrombosed IVC with partial refilling was seen, suggesting a hypoplastic IVC. Another potential cause of absent IVCs is renal thrombosis, a phenomenon encountered in unwell infants and has been shown to accompany IVC thrombosis and regression of the infra-renal IVC [14, 17]. The terminology ‘absent IVC’ could therefore be a misnomer, with ‘hypoplasia’ being potentially more appropriate.

While hypoplasia of an infra-renal IVC is a rare but well-documented anomaly, the CT in this case demonstrates a normal infra-renal IVC with a hypoplastic suprarenal IVC [4, 7]. This is a highly unusual presentation, with only one prior documented case [18].

To our knowledge, there are three reported cases of PE with associated IVC abnormalities [10–12]. The mechanism for DVT is peripheral venous stasis, rather than blood hypercoagulability [19]. PEs are secondary to clot formation within the collateral veins that travel through the heart to the lungs.

There were 62 documented cases of abnormal IVC presenting with DVT as of 2010 [20]. Approximately 5% of patients presenting with unprovoked DVT under the age of 30 were found to have IVC abnormalities, indicating that it is a significant risk factor [21]. Intense exercise is a trigger for DVT [20]. In our patient, we postulate that the congenital IVC abnormality was the major risk factor, but that the intense exercise could have contributed to the extent of the clot.

Current NICE guidelines do not require a CT for patients with DVT unless there is concern of cancer [ 22]. Without a CT, patients with absent IVCs as the cause of their DVT may go undetected. Due to the increased thrombotic risk, patients under the age of 30 with symptoms of bilateral DVT provoked by exercise must therefore be considered for imaging to investigate abnormal IVC development.
